# Time Course of Perceived Visual Distortion and Axial Length Growth in Myopic Children Undergoing Orthokeratology

**DOI:** 10.3389/fnins.2021.693217

**Published:** 2021-10-13

**Authors:** Guihua Liu, Yiyuan Wu, Hua Bi, Biying Wang, Tianpu Gu, Bei Du, Jianliang Tong, Bin Zhang, Ruihua Wei

**Affiliations:** ^1^Tianjin Key Laboratory of Retinal Functions and Diseases, Tianjin Branch of National Clinical Research Center for Ocular Disease, Eye Institute and School of Optometry, Tianjin Medical University Eye Hospital, Tianjin, China; ^2^College of Optometry, Nova Southeastern University, Davie, FL, United States; ^3^Doctor’s Exchange of Georgia PC, Lawrenceville, GA, United States

**Keywords:** orientation discrimination threshold, high-order aberration, visual distortion, axial length, orthokeratology

## Abstract

**Purpose:** To establish the time course of the subjective visual function changes during the first month of orthokeratology treatment in myopic children, and to investigate how the time course variations are associated with the objective optical quality changes and the axial length growth (ALG) after 1 year of treatment.

**Methods:** A total of 58 myopic children aged from 8 to 16 years participated in this self-controlled prospective study. All subjects were fitted with designed spherical four-zone orthokeratology lenses. Subjective visual function was evaluated with orientation discrimination threshold (ODT), and objective optical quality was quantified with the high-order aberration root-mean-square (HOA-RMS) and the changing speed of HOA. The measurements were done before the lens fitting and 1 day, 1-, 2-, and 4-weeks after lens wear. Axial length was obtained at baseline and 1-year follow-up, and ALG was defined as the difference. One-way ANOVA was conducted to compare the difference for statistical analysis.

**Results:** After lens fitting, the ODT time courses peaked on day 1 in 28 children, 1 week in 15 children, 2 weeks in 11 children, and 4 weeks in 4 children. In contrast, the HOA-RMS steadily rose during the first month, and the changing speed of HOA was only transiently elevated on day 1 after the initial lens wear. The ALG was 0.12 ± 0.20 mm in subjects whose ODT peaked at day 1, 0.08 ± 0.09 mm in subjects whose ODT peaked on 1-week, and 0.12 ± 0.15 mm in subjects whose ODT peaked on 2-week or later. There was no difference in axial growth among the subjects whose ODT peaked at different days (*P* = 0.734).

**Conclusion:** While half ODT time course resembled the changing speed of HOA with a transient elevation on day 1, about a quarter of the ODT time course resemble the steadily rising of HOA-RMS, and the rest was located in the middle. The ALGs in children with different types of ODT time courses were similar.

## Introduction

The prevalence of myopia has dramatically elevated over the past several decades, especially in East Asian countries and regions (Pan et al., 2015; Holden et al., 2016), where up to 90% of teenagers and young adults in China and 96.5% of 19-year-old men in Seoul are myopic (Dolgin, 2015). It is also one of the World Health Organization’s immediate concerns (Fricke et al., 2012). Among optical methods developed to retard myopia progress, orthokeratology is one of the most often applied devices in clinics. It is a rigid contact lens with a reverse geometry on its back surface. It alters the shape of the front corneal surface through overnight wearing (Nichols et al., 2000). During the daytime, the cornea’s flattened central portion reduces refractive errors and improves visual acuity (Swarbrick, 2006). The steepened mid-peripheral cornea can induce a myopic defocus shift on the retina, which may be the underlying mechanism for axial growth retardation. Orthokeratology lens can reduce 32–63% per year in subjects of different ethnicities (Charm and Cho, 2013; Li et al., 2017; Santodomingo-Rubido et al., 2017).

With an altered front optical surface, the OK lens also leads to increased optical aberrations and straying light in the patients undergoing such treatment (Hiraoka et al., 2007; Liu et al., 2017). Higher-order aberrations sharply increased 7 days after lens wearing and remained high when measured 1-year treatment (Stillitano et al., 2008; Santolaria Sanz et al., 2015; Santolaria-Sanz et al., 2016; Xia et al., 2020). However, patients’ visual performances wearing orthokeratology lenses were surprisingly good. In adult patients aged 18–43, the perceived visual distortion was measured by reporting if the briefly turn-on LED lights were visible in the peripheral visual field. It transiently increased 1 day after lens fitting and returned to baseline level 1 week after lens wearing (Santolaria Sanz et al., 2015; Santolaria-Sanz et al., 2016). In children aged between 8 and 14, the threshold to detect shape distortion was not significantly different from the baseline level at 1 week and 1 month after the lens wear (Xia et al., 2020). Rapid neural adaptation was proposed to explain the disparity between subjective visual function and optical quality.

However, several vital questions remained unclear. Firstly, the adult study reported the average trend of light distortion perceived and did not disclose the variance among individuals’ time courses. Since it is unlikely that everyone returned to baseline level at 1 week, it would be more informative by revealing the time course variations. Secondly, the children’s study had the first follow-up visit scheduled 1 week after the initial lens wear. It is not clear if the transient elevation in light distortion reported in adults also exists in children or only in a part of the children. More importantly, neither study provided the axial growth data. The children’s research stopped at 1 month. Although the adult study had a 1-year follow-up, the author did not report the axial length growth probably because axial length in adults was insignificant. It is unclear if the variations in light distortion time course are associated with differential axial length growth.

In the present study, we aimed to track the changes in visual distortion with the orientation discrimination threshold (ODT) method during the first month of lens-wear and measure the axial length growth over a year. Moreover, we attempted to clarify if the variations in light distortion time course are associated with axial length growth.

## Materials and Methods

### Subjects

This self-controlled prospective study was conducted at the Tianjin Medical University Eye Hospital (Tianjin, China) between September 2019 and November 2020. The inclusion criteria were: aged between 8 and 16 years; myopia greater than −0.75 D and less than −5.00 D; with-the-rule astigmatism less than −1.5 D; best-corrected monocular optical acuity better than 20/20; no strabismus or ocular surface disease; no history of surgery or contact lens wear. This study protocol adhered to the Declaration of Helsinki’s tenets and was approved by the Ethics Committee of Tianjin Medical University Eye Hospital. After being informed of the potential risks related to this study, all subjects and their guardians signed informed consent forms before any procedures were performed. Only right eye data were taken into analysis to avoid the interference caused by the interocular correlation.

### Measurements

Uncorrected visual acuity, subjective refraction, high-order aberration, and orientation discrimination threshold (ODT) tests were measured before the lens fitting and 1 day, 7 days, 14 days, and 1 month after lens dispatch. Axial length was measured at baseline and 1-year follow-up.

### Orthokeratology

Subjects were fitted with spherical four-zone orthokeratology lenses (Euclid Systems Corporation, Herndon, United States) composed of oprifocon A (Boston EQUALENS II) with an oxygen permeability (DK) of 85 × 10^–11^ (cm^2/^s) (mL O_2_/mL mmHg). Total lens diameter ranged from 10.6 to 10.8 mm, the back optic zone diameter (BOZD) was 6.0 mm, and the reverse curve (RC) width was between 0.6 and 0.8 mm. The lens fitting procedures strictly followed the guidelines provided by the lens manufacturer. In brief, lenses were first selected based on the measurements of a subject’s horizontal visible iris diameter (Lenstar LS900; Haag-Streit AG, Bern, Switzerland), the flat-*K* value of the 3 mm zone, and corneal eccentricity value (Medmont E300; Medmont International Pty., Ltd., Nunawading, VIC, Australia). Fitting quality was evaluated by fluorescence staining 1 h after the lens dispatch. A good fitting was indicated by an optical zone coving the pupil, no apparent decentration of the lens, lens movement less than 1 mm with blinks, and a bulls-eye pattern with fluorescence staining. After lens delivery, subjects were required to wear the lens for more than 8 h per night and at least 6 days per week.

### High-Order Aberration

**FIGURE 1:**
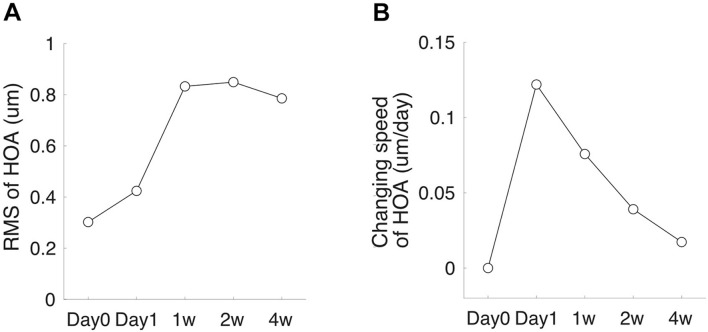
High-order aberration (HOA) measurement schedule. **(A)** A subject’s HOA-RMS. **(B)** The subject’s changing speed of HOA at each follow-up visit.

Corneal topography was obtained with Pentacam (OCULUS Optikgeräte GmbH, Wetzlar, Germany) (Pinero et al., 2009; Greenstein et al., 2012; Rudolph et al., 2012; Gyldenkerne et al., 2015). The system’s internal software automatically decomposed the corneal elevation data into Zernike polynomials up to the 10th order (Pinero et al., 2009). The root-mean-square (RMS) of total higher-order aberrations (HOA, 3rd–6th orders) aberrations were extracted from the central corneal zones with a diameter of 6 mm. During the measurement, the Pentacam uses a blue Light Emitting Diode (LED) slit beam to illuminate a cornea and anterior chamber. The subject was instructed to fixate on a target in the center of the slit beam. Three scans were taken per eye. Each scan was automatically assigned a quality score by the system, and only the scan with an acceptable score was taken into analysis. Low-quality scans were deleted, and the measurements were repeated. The changing speed of HOA-RMS was calculated as the difference between the two follow-up visits divided by the number of days between the two visits (Figure 1).

### Orientation Discrimination Threshold Test

Orientation discrimination threshold test stimuli were presented on a 24.1-inch (48 cm × 26 cm) screen (AOC22732, 1920 × 1080 pixels, 60 Hz) at a viewing distance of 1 m. Each pixel subtended a visual angle of 0.66 arcmins horizontally and vertically. All patients were tested monocularly as the non-tested eye occluded with an opaque patch. The tested eye’s distance refractive error was corrected if the visual acuity was less than 20/20. Before each trial, a subject fixated at the center of a 4-degree-diameter ring, within which test stimuli were presented. Following a button press, the ring disappeared and, after a 1 s delay, the first stimulus pattern was presented for 50 ms, followed by a 200 ms blank interval. The fixation ring then reappeared for 50 ms; the second stimulus pattern was presented 1 s later for 50 ms and was followed by a 200 ms blank interval. The duration of each pattern was set at 50 ms to minimize the effect of scanning eye movements (Fu et al., 2017; Chen et al., 2018). All ODT tests were performed with full optical correction to remove the lower order aberrations, including defocus and astigmatism.

**FIGURE 2:**
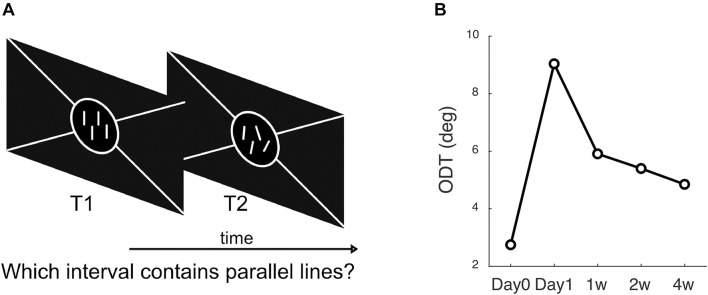
Methods to measure ODT. **(A)** The subject’s task was to answer which interval contains the parallel lines, 1st or 2nd. **(B)** A subject’s ODT measured at each follow-up visit.

One stimulus pattern presented at each trial consisted of four bright parallel lines, each of which was two pixels (1.3 arcmin) wide and 0.4° in length, presented vertically on a dark background. Each line was randomly located inside the preceding fixation ring, and the distance between two adjacent lines was at least 0.5° to prevent overlap. The other stimulus pattern had the same arrangement except that the orientations of the four lines were randomly deviated from the vertical direction according to a standard deviation (SD). At each trial, the subject had to decide which pattern contained the parallel vertical lines. The order of the parallel and non-parallel line patterns varied randomly from trial to trial (Figure 2).

The test was programmed in MATLAB (MathWorks, Natick, MA, United States) and Psychophysics Toolbox version 3 (Brainard, 1997). A Bayesian adaptive two-alternative forced-choice task based on the ZEST algorithm was used (King-Smith et al., 1994). From trial to trial, the program adjusted the non-parallel lines’ SD based on the subject’s previous responses. A Weibull function with a slope of 3.5 was used as the likelihood function in the ZEST algorithm. The initial angular SD was set at 8° according to values from a previous study. After each trial, the probability density function (PDF) of the experimentally determined angular SD was updated by multiplying the likelihood function with the prior PDF until the confidence interval for the PDF was less than 0.3 log units or a total of 30 trials were finished. The output SD, which was equal to the final PDF’s mean, was defined as the ODT. Testing of each eye required about 2 min to finish.

Each subject’s ODT was measured three times at each visit, and the average value was taken into analysis. The test-retest reproducibility was quantified with the intraclass correlation coefficient (ICC), and a value greater than 0.75 indicated good reliability (Chen et al., 2018).

### Axial Length

Axial length was measured with a non-contact biometer (Lenstar LS-900; Haag-Streit AG, Bern, Switzerland). Subjects were asked to keep both eyes open and fixate on the target. Between measurements, subjects blinked multiple times to ensure an intact tear film to minimize potential measurement errors. Five measurements were taken for each axial length measurement, and only the repeats with intra-session differences less than 0.02 mm were averaged and recorded.

### Statistical Analysis

The means and standard deviations of ODT, HOA-RMS, HOA changing speed, axial length growth were computed for descriptive purposes. One-way ANOVA was used to compare if the differences among groups were significant. All statistical analyses were performed using R software (version 3.2.2)^1^. A *p*-value < 0.05 value was defined as statistically significant.

## Results

**FIGURE 3:**
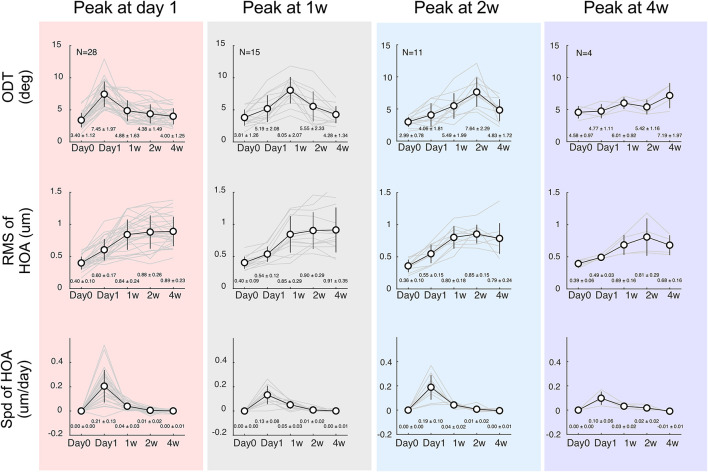
Time courses separated according to the ODT peaking time. Top row: discrimination threshold (ODT), Middle row: Residua Mean Square (RMS) of high-order aberrations (HOA), Bottom row: changing speeding of HOA. The left column shows subjects whose ODT peaked at day 1 (red shade); the middle column shows subjects whose ODT peaked at 1 week (gray shade), and the right two columns show subjects whose ODT peaked at 2 weeks (blue shade) or 4 weeks (purple shade), respectively.

Among a total of 65 subjects who were enrolled initially, 58 completed all follow-up examinations [30 males and 28 females, mean age 12.10 ± 2.01 years (range: 8 –16 years)]. All participants were first categorized according to the time at which ODT peaked. In 28 children, ODT reached the peak 1 day of OK lens wear and declined afterward. Fifteen children had ODT reaching the peak at 1 week and decreased thereafter. In 11 and 4 children, the ODT reached the peak at 2 weeks and 4 weeks after the initial lens wearing, respectively (top tows, Figure 3). However, regardless of the peaking time of ODT, HOA-RMS increased steadily during the first month after lens wear (middle row, Figure 3). The changing speed of HOA was transiently elevated on the first day after the lens wear and immediately declined afterward (bottom row, Figure 3).

**FIGURE 4:**
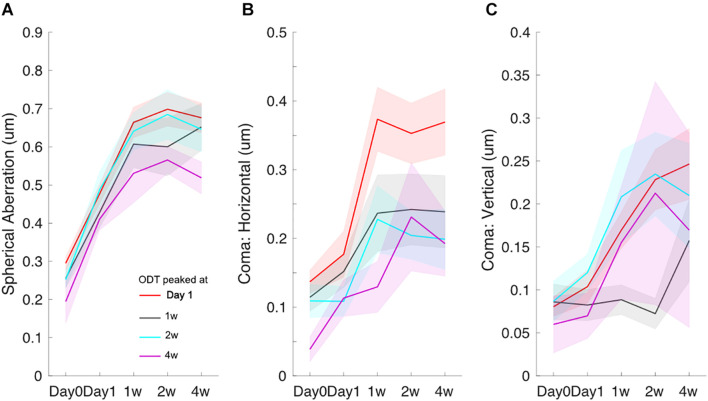
Time courses of spherical aberration **(A)**, horizontal coma **(B)**, and vertical coma **(C)**. Redline: subjects whose ODT peaked at day 1, black line: subjects whose ODT peaked at 1 week, blue line: subjects whose ODT peaked at 2 weeks, and purple line: subjects whose ODT peaked at 4 weeks. The shaded area indicates mean ± one standard error.

The time course for spherical aberration (Figure 4A), coma horizontal (Figure 4B), and coma vertical (Figure 4C) did not appear to match the ODT time course. Spherical aberration significantly increased in all subjects and reached the peak mostly around 2 weeks (one-way ANOVA, *p* < 0.001 in all four groups). Horizontal coma component significantly increased in the subjects whose ODT peaked at day 1 (one-way ANOVA, *p* < 0.001). However, the peak value of horizontal coma was reached at 1 week and remained high afterward. Horizontal coma in other groups showed increased trend, but not reaching significant level [one-way ANOVA, *p* = 0.15 for subjects whose ODT peaked at 1 week (black), *p* = 0.06 for subjects whose ODT peaked at 2 weeks (blue), and *p* = 0.08 for subjects whose ODT peaked at 4 weeks (purple)]. Similar patterns were found in vertical coma component.

**FIGURE 5:**
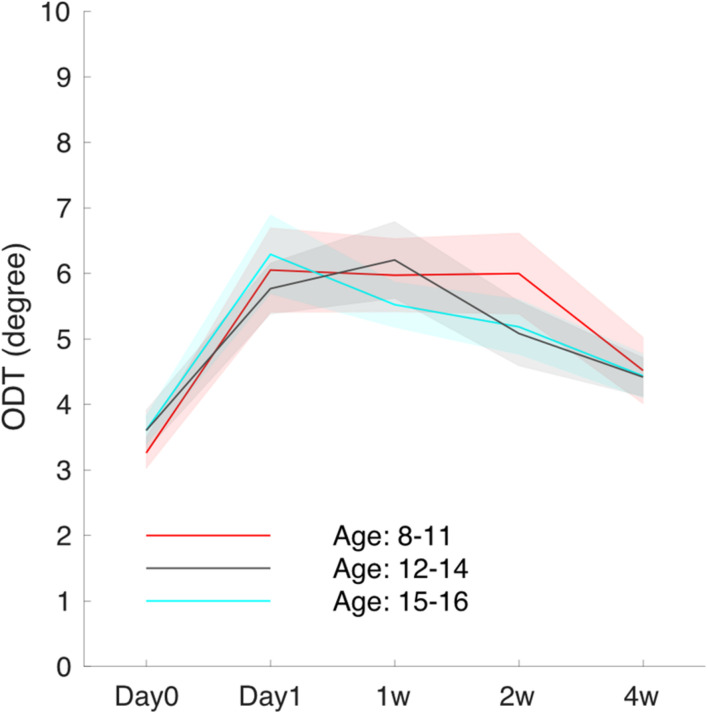
The averaged ODT time course for subjects grouped by age.

To explore the effect of age on the ODT time course, subjects were separated into three age groups (8–11 years, 12–14 years, and 15–16 years), and the mean ODT time courses were computed (Figure 5). Subjects in the youngest group and oldest group both reached the peak on day 1.

**FIGURE 6:**
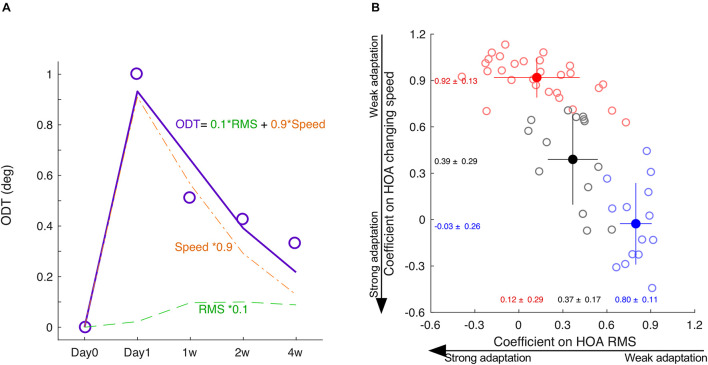
The decomposition of ODT time course. **(A)** The ODT time course is decomposed as a linear combination of the time courses of HOA-RMS and HOA changing speed. **(B)** The distribution of coefficients of subjects peaked at day 1 (red), day 7 (black), and at or after day 14 (blue). SPD, speed. * means multiplication.

In each subject, we normalized the HOA-RMS and the changing speed of HOA to its peak value, respectively. Normalization did not change the shape of the time courses, and there was no significant correlation between the two normalized indexes (*r* = −0.24, *p* = 0.09) population-wise. Therefore, the ODT time course could be decomposed as a linear combination of the time courses of the normalized HOA-RMS and the changing speed of HOA (Figure 6A). The subjects whose ODT peaked at 2-week or later had greater coefficient values in HOA-RMS and small coefficient values in HOA changing speed (Blue dots, Figure 6B). Those subjects’ ODT time courses had a high resemblance to the HOA-RMS time course and were less parallel to the HOA changing speed time course. In other words, they may have weak adaptation to HOA-RMS and strong adaptation to HOA changing speed. The subjects whose ODT time course peaked at day 1 had a small coefficient on HOA-RMS and a large coefficient value on HOA changing speed. Their ODT time courses had less resemblance to the HOA-RMS time course and were more parallel to the HOA-changing speed’s time course. In other words, they may have a strong adaptation to HOA-RMS and a weak adaptation to the HOA changing speed (Red dots, Figure 6). The subjects with ODT peaked at 1 week were located in the middle and had similar coefficient values to either HOA-RMS or HOA changing speed (Black dots, Figure 6).

**FIGURE 7:**
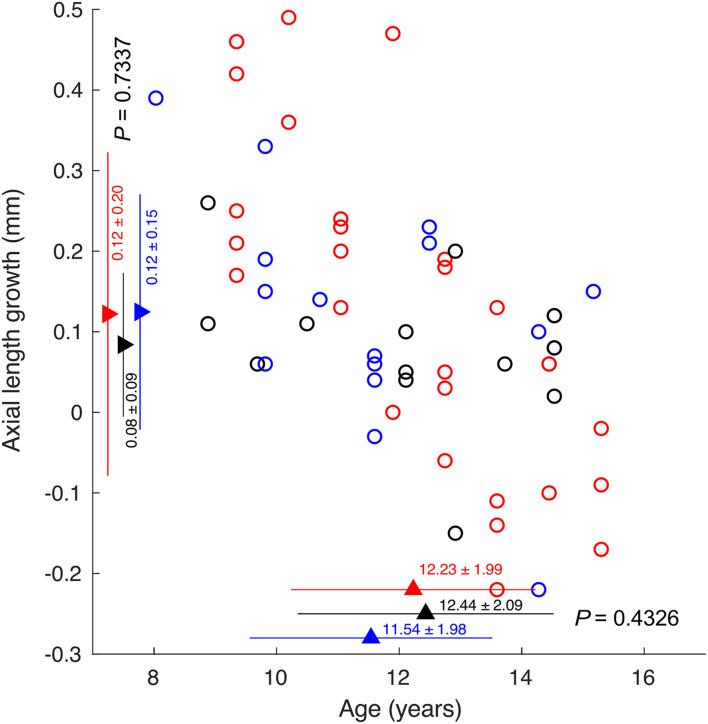
The correlation between axial length growth and age. Circles indicating subjects whose ODT peaked at day 1 (red), 1 week (black), and 2 weeks or later (blue).

The axial length growth was significantly correlated with baseline age (*r* = −0.61, *P* < 0.001). However, there was no difference in axial growth among the subjects whose ODT peaked at different days (one-way ANOVA, *P* = 0.73). There was also no difference in age among the subjects who showed ODT peak at different days (One-way ANOVA, *P* = 0.44) (Figure 7).

## Discussion

The most critical finding in the present study is ODT time courses’ variations. Instead of uniformly peaking 1 week after the initial lens-wear, about half of the subjects experienced a transient increase in ODT, a quarter reached the peak at 1 week, and a quarter peaked relatively late at 2 weeks or later. In contrast, the time course of HOA-RMS and HOA changing speed were quite similar across different subjects. HOA changing speed only had a transient increment on day 1, and the HOA-RMS time course steadily rose during the first month. We proposed that the ODT time course could be interpreted as a combination of the time courses of HOA changing speed and HOA-RMS, and neural adaptation plays an important role underlying the variance in the ODT time course. The other essential finding was that ODT time courses’ variations were not associated with axial length growth and age.

### Orientation Discrimination Threshold Peaking After 1 Week

One key difference between the present study and previous studies was that half of the subjects had ODT peaking at 1 week or later. Previous studies done in both adults and children reported no changes in visual performance 1 week after the initial lens-wear (Santolaria Sanz et al., 2015; Santolaria-Sanz et al., 2016; Xia et al., 2020). This study captured the ODT peaking both before and after 1 week of lens wear. The difference could partially be attributed to the different methods used to evaluate subjective visual distortion. The human visual system may utilize two mechanisms to minimize distortion perception. When the information sent from a retina region is distorted or missing, the central visual system can take the information from surrounding regions into processing to “fill in” or complete the visual scene (Ramachandran and Gregory, 1991; De Weerd et al., 1995). Moreover, due to the relatively long stimulus presentation duration, subjects may not maintain steady fixation throughout the examination (Tarita-Nistor et al., 2012, 2017). Unstable fixation may “average” all distorted or missing lines over time. The spatial and temporal redundancy embedded in the methods used by previous studies facilitates the process of “filling-in” and “averaging.” In the children’s study, the visual stimulus used was a radial pattern covering 360°, making the filling-in easier (Xia et al., 2020). The visual stimulus used in the adult study was not static. Instead, it moved from the periphery toward the center along the pre-defined semi-meridians, which increased the possibility of unstable fixation. The stimulus presentation duration was 250–750 ms in the adult studies (Santolaria Sanz et al., 2015; Santolaria-Sanz et al., 2016) and 500 ms in the children study (Xia et al., 2020). That long fixation time allows for unstable eye fixation and eye movements. Such two processes can mask the early and subtle functional changes until the function’s loss becomes sufficient to exceed the threshold. For the ODT test used in this present study, a short and randomly distributed set of line segments was used to minimize the “fill in” phenomenon (Williams et al., 1984). The present study’s stimulus duration was only 50 ms, too short to trigger saccadic eye movements. Such a design minimizes the possibility of “average.”

### Variations in Orientation Discrimination Threshold Time Course and Adaptation

When the environment changes, the visual system’s adaptation is a mechanism to achieve satisfactory performance (Clifford et al., 2007). Adaptation has been displayed in light (Matesanz et al., 2011; Takemura et al., 2020), color (Eisner and Enoch, 1982; Neitz et al., 2002; Delahunt et al., 2004; Belmore and Shevell, 2008, 2011), contrast (Kwon et al., 2009; Bao and Engel, 2012; Bao et al., 2013), distortion and blur (Adams et al., 2001; Yehezkel et al., 2010; Habtegiorgis et al., 2017, 2018). The significantly increased high order aberrations reported in this study were consistent with previous studies on orthokeratology lens (Santolaria Sanz et al., 2015; Santolaria-Sanz et al., 2016; Xia et al., 2020). Here, we propose that neural adaptation may have played an essential role in the emergence of different types of time courses of ODT. HOA’s changing speed was only significantly elevated on day 1, while the HOA-RMS steadily rose during the first month following lens-wear. The subjects whose ODT peaked at day 1 did not adapt well to the transient elevation in the changing speed of HOA and adapted well to the steadily rising HOA-RMS. This may explain the large coefficient in changing speed of HOA and the small coefficient in HOA-RMS. On the other extreme, the subjects whose ODT peaked at 2 weeks or later adapted well to the transient elevation in HOA changing speed but were slow at adapting to the steadily rising HOA-RMS. This may explain the small coefficient in changing speed of HOA and the large coefficient in HOA-RMS. With only four subjects peaking at 4-week, the steadily rising HOA-RMS adaptation was mostly completed by the end of the first month. This was consistent with a previous study showing that the lack of adaptation to image distortion is the cause of long-term visual discomfort for some novices with progressive extra glasses (Meister and Fisher, 2008). The 15 subjects whose ODT peaked at 1 week were mixed cases in the middle, with relatively balanced adaptation to either the transient elevation of HOA changing speed and steadily rising HOA-RMS.

### No Correlation Between Orientation Discrimination Threshold Time Course and Axial Length Growth

The second vital finding from this present study was that axial length growth was not different in the three different categories of subjects. This indicates that whether a subject can adapt to the HOA-RMS or HOA change is not influential in determining the axial growth. This result was consistent with the findings that an intact retina-brain link was not required for axial growth. In chicks with optic nerve sectioned, optically occluded eyes demonstrated longer axial growth than those of non-occluded eyes (Troilo et al., 1987). Compensation to hyperopic defocus was found in chicks with either or both optic nerve section (ONS) and ciliary nerve section (CNS; Miles and Wallman, 1990; Diether and Schaeffel, 1997; Wildsoet, 2003). The myopia progression depends on retina local cues (Wallman et al., 1987). Such a mechanism was also confirmed in primates (Smith et al., 2009; Read et al., 2010).

In this study, the subjects who peaked at different days had no age difference. This is in contrast to the findings in other previous studies. For example, Li reported that younger children might have an easier or faster adaptation to the lenses, which could compensate for the optical disturbance induced by lenslets while looking through the lenses’ peripheral parts. Better adaptation of blur and acceptance of the lenses were also found in younger children wearing Defocus Incorporated Multiple Segments (DIMS; Lu et al., 2020). Adults were more sensitive to the visual symptoms by reporting headaches and dizziness after wearing DIMS. Furthermore, in contrast to 85% of the children who were willing to wear DIMS lenses, the acceptance rate in adults was only 60% due to unbearable discomfort. Chang and Cheng (2020) reported decreased contrast sensitivity resulting from optical and neural components of visual processing and revealed a greater extent in adults than children after wearing orthokeratology for 28 days.

### Limitations of Our Study

This present study has several limitations. First, we only measured cornea HOA, but the whole-eye HOA. With orthokeratology lens alters the front surface of the cornea, the difference between these two types of HOA should be slight. Second, both HOA measurement and ODT test were done in monocular conditions, which is different from the daily use of binocular vision (Brito et al., 2015; Santolaria Sanz et al., 2015). Third, the association between ODT values and the subjects’ visual distortions, such as ghosts, haloes, and starbursts, is unclear. Fourth, we did not evaluate other factors contributing to visual distortion, such as contrast sensitivity changes (Santolaria Sanz et al., 2015; Santolaria-Sanz et al., 2016) and pupil size (Chen et al., 2012).

### Clinical Significance

The time course revealed from this present study provided guidance on expecting visual discomforts reported by children who received orthokeratology. Half of the complaints would be anticipated at day 1, and the majority of the visual discomforts should be reported within the first week. Any visual discomforts reported beyond 2 weeks deserve more attention from the clinicians. Recently, it has been reported that persons with different personalities may have different sensitivity to image quality (Woods-2010). People who lack confidence tend to hesitate at reporting blurred images and choose to endure them while waiting for more substantial evidence to emerge. Therefore, a questionnaire on personality would help predict the subjective complaints from the children undergoing orthokeratology. Meanwhile, the clinician should have confidence in the outcome of orthokeratology. Even a patient who has late visual discomfort can achieve good axial growth retardation.

## Conclusion

In children who underwent orthokeratology treatment, their ODT significantly increased during the first month after the initial lens wear. While 25% of the children, the ODT time course resembles the HOA-RMS, which showed a steady increase, about half ODT time course resemble the changing speed of HOA, which was most significant during the first day. Children with different types of ODT time courses showed similar axial growth.

## Data Availability Statement

The original contributions presented in the study are included in the article/supplementary material, further inquiries can be directed to the corresponding authors.

## Ethics Statement

The studies involving human participants were reviewed and approved by the Ethics Committee of Tianjin Medical University Eye Hospital. Written informed consent to participate in this study was provided by the participants’ legal guardian/next of kin.

## Author Contributions

BZ, RW, and GL conceived the experiments. HB, GL, and JT determined the experimental methods. YW, TG, and BD performed the experiments. BZ and GL analyzed and interpreted the data. BW and GL wrote the manuscript. BZ and RW modified the manuscript. All authors contributed to manuscript revision, read, and approved the submitted version.

## Conflict of Interest

JT hold patents (8092025, 7806528) on the ODT described in this article. The remaining authors declare that the research was conducted in the absence of any commercial or financial relationships that could be construed as a potential conflict of interest.

## Publisher’s Note

All claims expressed in this article are solely those of the authors and do not necessarily represent those of their affiliated organizations, or those of the publisher, the editors and the reviewers. Any product that may be evaluated in this article, or claim that may be made by its manufacturer, is not guaranteed or endorsed by the publisher.

## Footnotes

1
http://www.R-project.org/
